# Multiple Job Holding, Job Changes, and Associations with Gestational Diabetes and Pregnancy-Related Hypertension in the National Birth Defects Prevention Study

**DOI:** 10.3390/ijerph21050619

**Published:** 2024-05-14

**Authors:** Amel Omari, Miriam R. Siegel, Carissa M. Rocheleau, Kaori Fujishiro, Kristen Van Buren, Dallas Shi, A.J. Agopian, Suzanne M. Gilboa, Paul A. Romitti

**Affiliations:** 1 Division of Field Studies and Engineering, National Institute for Occupational Safety and Health, Cincinnati, OH 45213, USAopv7@cdc.gov (K.V.B.);; 2 Epidemic Intelligence Service Officer, Centers for Disease Control and Prevention (CDC), Atlanta, GA 30329, USA; 3 Department of Epidemiology, Human Genetics, and Environmental Sciences, UTHealth School of Public Health, Houston, TX 77030, USA; 4 Division of Birth Defects and Infant Disorders, National Center on Birth Defects and Developmental Disabilities, Centers for Disease Control and Prevention (CDC), Atlanta, GA 30329, USA; 5 Department of Epidemiology, College of Public Health, The University of Iowa, Iowa City, IA 52242, USA; paul-romitti@uiowa.edu

**Keywords:** gestational diabetes, hypertensive disorders of pregnancy, job change, maternal morbidity, multiple-job holding

## Abstract

We used National Birth Defects Prevention Study data to investigate associations between working patterns shortly before and during pregnancy and gestational diabetes and pregnancy-related hypertension. We analyzed working patterns (multiple-job holders, job changers, single-job holders) during the three months before and during pregnancy for 8140 participants who delivered a live-born child without a birth defect. “Multiple-job holders” worked more than one job simultaneously, “job changers” worked more than one job with no overlap, and “single-job holders” (referent) worked one job. We used multivariable logistic regression to estimate associations between working pattern and each outcome, adjusting for maternal age and educational attainment at delivery. We explored effect measure modification by household income, peak weekly working hours, and maternal race/ethnicity. Multiple-job holders had higher odds of gestational diabetes (adjusted odds ratio [aOR]: 1.5; 95% confidence interval [CI]: 1.1–2.1) and pregnancy-related hypertension (aOR: 1.5; 95% CI: 1.0–2.2) compared with single-job holders. Multiple-job holders with a household income of more than 30,000 USD per year, 32–44 peak weekly working hours, and from racial/ethnic minority groups had higher odds of gestational diabetes compared with single-job holders in respective categories. Detailed occupational information is important for studies of occupation and maternal health.

## 1. Introduction

Work is understudied as a social determinant of health [1]. Despite high workforce participation among people of reproductive age [2], how pregnant workers’ labor force engagement might be associated with maternal health is not clear. This might be due, in part, to how previous studies have analyzed measures of only a single, “main” job [3].

This study examines rarely-explored relationships between changing jobs and holding multiple simultaneous jobs during pregnancy with respect to maternal outcomes [4], both likely relatively common working patterns among pregnant workers [5]. We use the term “working patterns” to refer to how pregnant workers engage in the paid workforce, including multiple-job holding, job-changing, and single-job holding during preconception and pregnancy. Non-standard work arrangements (such as contract or freelance work, on-call workers, or day-laborers) represented about 10% of workers in 2017 according to the Current Population Survey [6], and such arrangements may be associated with holding multiple jobs [3]. Pregnancy is also associated with family-related job changes [5].

The relationship between working hours and pregnancy-related hypertension or pre-eclampsia, and with gestational diabetes mellitus (GDM), is poorly understood and, to our knowledge, no prior work has accounted for differences in patterns between people who work long hours in a single job versus across multiple jobs [7]. This may be in part because of measurement limitations in large surveys. Surveys often ask participants about a single, “main” job [3,8,9]. Some health surveillance systems collect information about jobs worked in the last week or month, missing measurement of changes in employment during a worker’s pregnancy [8,10]. The present study adds nuance to understandings of employment conditions by accounting for holding multiple jobs or changing jobs, and in this way might help clarify inconclusive findings in the health literature (we use the term “employment conditions” to refer to “the formal and informal arrangements between workers and employees that determine both contractual (e.g., wages, hours) and relational (e.g., participation in decision-making, power dynamics) components of one’s job” [11]).

GDM and pregnancy-related hypertension are relatively common and associated with future adverse maternal health outcomes [12,13]. Prior work on holding multiple jobs and other health outcomes [14,15] suggests two main mechanisms that might link holding multiple jobs with health outcomes through stress, both of which might be relevant for maternal morbidities: time constraints and potential for poor employment quality. Managing schedules across multiple jobs could impact workers’ experience of role conflict and strain, which might associate with stress exposure [16,17]. Employment quality is a multidimensional concept that may include such considerations as “employment stability (e.g., the type and length of contract), material rewards (e.g., pay and benefits), working time arrangements (e.g., the length and predictability of work hours),” and others [11,18]. Peckham and colleagues (2022) drew on prior work [19,20] to define seven dimensions of employment quality: employment stability, material rewards, workers’ rights and social protection, working time arrangements, employability opportunities, collective organization, and interpersonal power relations. Such employment quality-related considerations have been conceptualized as sources of stress with relevance for a range of negative health outcomes in prior work [21], including adverse birth outcomes [22]. If multiple-job holders have poorer-quality employment, this could lead to higher stress and difficulty accessing resources that are protective to maternal health, such as high-quality nutrition, health insurance, and others. Indeed, prior work found that among low-income mothers of young children, multiple-job holders were more likely to work non-standard schedules and long hours compared to single-job holders [15]. Thus, we hypothesized that multiple-job holders would have higher odds of GDM and pregnancy-related hypertension diagnoses.

Job changing has rarely been examined with relation to maternal health beyond the assessment of job loss as part of stress indices, despite being relatively common among pregnant workers [5,23,24]. Job changing might be an independent source of stress for pregnant workers, and job changing might block access to benefits that activate after one year of tenure for some workers [25]. For these reasons, we hypothesized that job changers would have higher odds of GDM and pregnancy-related hypertension diagnoses.

In the current study, we compared characteristics of workers who (1) held multiple jobs simultaneously during the three months pre-conception through the end of pregnancy and (2) changed jobs during this same time period with those who held a single job during the same period. We used data from a population-based sample of mothers who delivered live-born infants without birth defects participating in the National Birth Defects Prevention Study to evaluate how holding multiple jobs and changing jobs in preconception and pregnancy relate to GDM and pregnancy-related hypertension. To explore heterogeneity in these factors, we further assessed how the relationship between these working patterns and outcomes changes by income level, working hours, and maternal race/ethnicity.

## 2. Materials and Methods

We used data from the National Birth Defects Prevention Study (NBDPS), which recruited women delivering children with birth defects (case individuals) and live-born children without birth defects (control individuals), recruited from a random sample of hospital delivery logs or birth certificates at NBDPS study sites [26,27]. Only data for maternal respondents of control children without birth defects delivered from 1 October 1997 through 31 December 2011 were analyzed. Respondents who did not work for pay during the study period (including students and homemakers) were excluded [28]. All interviewed study participants provided informed consent. The Centers for Disease Control and Prevention Institutional Review Board (IRB), along with the IRBs for each participating site, have approved the NBDPS (see 45 C.F.R. part 46; 21 C.F.R. part 56).

For pregnancy-related hypertension, only respondents interviewed after an updated survey launched in 2006 were analyzed. The updated survey asked specifically about pregnancy-related hypertension, whereas the original interview question did not differentiate between pregnancy-related hypertension and other types of hypertension.

GDM was operationalized as self-reported GDM diagnosis during the index pregnancy (assigned a 1), with reference to respondents with no diabetes diagnosis at any time (assigned a 0). Respondents who self-reported pregnancy-related hypertension, with or without pre-eclampsia or eclampsia, when they were pregnant with the index pregnancy were identified as having pregnancy-related hypertension. Reference respondents for the pregnancy-related hypertension analysis were never told by a doctor that they had high blood pressure, toxemia, pre-eclampsia, or eclampsia and did not have pregnancy-related high blood pressure during the index pregnancy. Although these NBDPS variables have not been validated due to the absence of a gold-standard source (e.g., medical records), other studies have found reasonable validity of self-reporting compared to medical records [29,30,31].

For the independent variable, respondents were asked about their work during the period from 3 months before estimated date of conception until the end of their pregnancy (hereafter, the “reporting period”). Respondents who reported working more than one job simultaneously during any time over the reporting period were defined as multiple-job holders. Respondents who reported working more than one job without overlap during the reporting period were defined as job changers. Respondents who held precisely one job during the reporting period were defined as single-job holders. We evaluated income, weekly working hours, and race/ethnicity as potential effect modifiers. Respondents were asked if their total household income in the year before the index pregnancy was less than 10,000 USD, 10,000–20,000 USD, 20,000–30,000 USD, 30,000–40,000 USD, 40,000–50,000 USD, or more than 50,000 USD. Income was dichotomized to group respondents with less than the sample median of 30,000 USD of prior-year household income and those with 30,000 USD or more. Peak weekly working hours were measured as the highest hours-per-week a respondent reported working at any time during the reporting period, summed across all reported jobs. Prior studies of long working hours and maternal health outcomes have grouped working hours in a range of ways [32,33,34,35]. We selected cutoffs to make strata with approximate part-time (<32 h per week), full-time (32–45 h per week), and long working hours (>45 h per week). Maternal racial/ethnic category was self-reported. Due to low cell counts in some race and ethnicity categories, those who reported their race/ethnicity in a category other than non-Hispanic White, non-Hispanic Black, or Hispanic were dropped from the effect measure modification analysis.

Because holding multiple jobs and changing jobs among pregnant workers is not well-understood, we assessed distributions of a range of factors thought to be meaningful to maternal health across working patterns. Pre-pregnancy maternal body mass index (BMI) was calculated from reported height and pre-pregnancy weight using National Institutes of Health cutoffs (underweight, normal weight, overweight, and obesity) ([36], NHLBI). Ever smoking during the period one month before estimated date of conception through the third month of pregnancy was categorized as a dichotomous yes/no variable. Whether the index pregnancy was the respondent’s first pregnancy (primigravida) or first live birth (primipara) was reported. Nativity and study site were also reported. Maternal age at delivery was measured as a continuous variable. Maternal educational attainment was categorized as less than high school, high school, some college, or college or more.

We conducted a descriptive analysis of all variables to summarize sociodemographic and other health-relevant variables across working patterns. Categorical characteristics were assessed for an association with the working pattern using chi-square, and *t*-tests were used to assess for an association between mean age and working pattern.

Using multivariable logistic regression models, we investigated associations between working pattern during pregnancy (holding multiple jobs or changing jobs compared to holding a single job) and two outcomes: GDM and pregnancy-related hypertension. Each outcome was analyzed separately. Prior study offers sparse guidance regarding whether the covariates that we analyzed descriptively might act as confounders or mediators of the relationship between working pattern and maternal health. We selected maternal age and educational attainment at delivery as factors with the strongest rationales for confounding this relationship [37,38,39,40,41,42,43] and adjusted all models for these two factors. Maternal self-reported smoking was not included as a confounding factor because it might instead act as a mediator of the relationship between working patterns and maternal morbidities, particularly if working patterns are differentially associated with stress exposure [44]. Although maternal BMI is often included in analyses of maternal health [45], chi-square analysis found no relationship between maternal BMI and working patterns; thus, it was not modeled as a confounder.

To explore possible effect measure modification as a secondary analysis, we conducted stratified adjusted multivariable logistic regression analyses, stratifying for household income, peak weekly working hours, and maternal racial/ethnic category, respectively. We conducted a sensitivity analysis using <10,000 USD, 10,000–50,000 USD, and >50,000 USD as cut points for our examination of household income as a potential effect measure modifier.

Finally, to increase our confidence in the temporal assumptions of this study (i.e., that the exposure precedes the outcomes), we conducted a sensitivity analysis that assessed respondents’ working pattern during only the period from 1 month before the estimated date of conception to 3 months after the estimated date of conception.

## 3. Results

Of 11,814 parents of infants without birth defects who participated in NBDPS, 8140 worked at least one job during the study period and were included in the analysis of GDM and working patterns. Of these, 3348 were asked the survey question specific to pregnancy-related hypertension and were included in the analysis of that outcome and working patterns.

Table 1 summarizes maternal characteristics by working pattern during the reporting period. Chi-square and *t*-tests showed significant differences or association for all descriptive variables, except for maternal BMI. Single-job holders were more likely than job changers and multiple-job holders to have higher household incomes (61%), have peak weekly working hours less than 32 per week (26%), report slightly older maternal age (mean = 28 years), and to be foreign-born (17%). Job changers were more likely than single and multiple-job holders to have lower household income (62%), report younger maternal age (mean = 25 years), report smoking during the periconceptual period through the first trimester (32%), and were less likely to have a college degree (25%). Multiple-job holders were more likely than single-job holders and job changers to work more than 45 h per week (63%) and have non-Hispanic White race/ethnicity (71%).

Multiple-simultaneous-job holders had 1.53 higher adjusted odds of reporting GDM compared to single-job holders (95% CI = 1.09–2.14) and 1.53 higher adjusted odds of reporting pregnancy-related hypertension compared to single-job holders (95% CI = 1.04–2.24) (Table 2).

Multiple-simultaneous-job holders with a household income of less than 30,000 USD had 1.72 higher odds of GDM (95% CI: 1.03–2.87), compared with single-job holders in the same income category (Table 3). Multiple-simultaneous-job holders working 32–45 h per week had roughly two and a half times higher odds of reporting GDM compared to single-job holders reporting peak weekly working hours in the same category (adjusted OR [aOR]: 2.61, 95% CI: 1.51–4.52) (Table 4). Finally, Hispanic multiple-job holders had 2.25 higher adjusted odds (95% CI: 1.09–4.65) of reporting GDM compared with single-job holders in the same racial/ethnic category (Table 5).

In our sensitivity analysis (Table S1, Supplementary Material), results varied after altering household income cut points to <10,000 USD, 10,000–50,000 USD, and >50,000 USD. Multiple-job holders with an income range of 10,000–50,000 USD had elevated odds of reporting GDM (aOR: 1.70, 95% CI: 1.04–2.77) compared to single-job holders in the same income category. Multiple-job holders in the lowest and highest income categories had elevated odds of reporting pregnancy-related hypertension (respectively: aOR: 4.24, 95% CI: 1.64–10.94, aOR: 2.14, 95% CI: 1.20–3.84) compared to single-job holders in the same income categories. Results of analyses restricted to mothers with jobs reported during the period of 1 month before estimated date of conception through the first trimester were similar to those reported from our main analysis.

## 4. Discussion

Our study explored the sociodemographic distribution of respondents across working patterns (holding multiple jobs, changing jobs, or holding a single job) experienced from three months before the estimated date of conception through the end of the pregnancy; associations between working patterns and GDM and pregnancy-related hypertension; and the potential effect measure modification of associations between working patterns and these maternal morbidities by selected sociodemographic characteristics. The results suggest that, compared to working a single job, working multiple jobs simultaneously during pregnancy may increase the risk for GDM and pregnancy-related hypertension. Multiple-job holders with a household income of <30,000 USD per year, 32–44 peak weekly working hours, and Hispanic ethnicity had higher odds of GDM compared with single-job holders in respective categories. We observed no associations between job change and outcomes.

This study contributes to the scarce literature on the relationship between holding multiple simultaneous jobs and maternal health. The single prior study we identified found no evidence of an elevated risk for adverse maternal health outcomes for Canadian mothers reporting holding more than one job per week during pregnancy using a non-specific maternal outcome questionnaire item [4].

Multiple-job holders may be heterogenous by the benefits versus demands of their holding multiple jobs [46]. If such heterogeneity exists in our sample, we speculate that estimates of an association between multiple-job holders and GDM and pregnancy-related hypertension may be conservative for multiple-job holders with higher exposure to factors such as low employment quality and/or high job demands [47]. Likewise, odds ratios may be overestimated for multiple-job holders with better employment quality. Lacking data to disaggregate multiple-job holders into classes as a prior study did [46], we used stratification to explore some of the heterogeneity we expected in working patterns, finding some evidence that there is variation in the relationship between multiple job holding and maternal morbidities.

The relationship between holding multiple jobs and GDM was increased only among those who worked 32–45 peak weekly working hours compared to single-job holders in the same working hours category. The absence of an association among those with long peak weekly working hours (>45 h) suggests that multiple-job holders with long working hours have similar odds of reporting GDM compared with single-job holders in the same working hours category. This diverges from Bruns and Pilskauskas’ [15] finding that showed an association between working long hours and depressive symptoms, possibly mediated by stress processes, among multiple-job holding mothers of young children. This work studied a different health outcome; it also analyzed a sample of low-income families. With this in mind, our finding that adverse health outcomes are not significantly associated with holding multiple jobs in the longest working hours category compared to single-job holders in the same working hours category could be due to an increased range of employment qualities in our sample. If multiple-job holders who work longer hours in our sample are likely to have jobs with higher employment quality (e.g., stability and sufficient benefits and income), the benefits of holding multiple jobs for these respondents may buffer the stress of working long hours at multiple jobs [17]; this is an empirically unstudied area ripe for future examination. On the other hand, if respondents in the standard working hours category (32–45 h/week) are disproportionately seeking multiple jobs to access adequate working hours (known as the “hours constraint” motivation [48,49]), respondents might be less likely to have the resources to buffer the stress of juggling multiple jobs, even with fewer hours. Replication with a larger sample size could increase confidence in this finding, and future studies could measure employment quality and job control among those working multiple or single jobs.

A stronger relationship between holding multiple jobs and GDM compared to holding a single job among lower household income respondents could be due to increased scheduling pressures, or lower employment quality among low-income multiple-job versus single-job holders [47]. Lower-income respondents may also be less likely to hold at least one job that offers healthcare or other benefits [50]. However, this pattern was not robust to changing cut points in the household income variable, and more research could help clarify the role that income plays in the relationship between holding multiple jobs and the outcomes we studied. Prior-year household income could also influence workers’ decision to take on additional jobs rather than functioning only as a marker of workers’ socioeconomic position. Finally, recall of prior-year income might be different for workers with non-standard work arrangements compared to workers with traditional employment and/or stable year-over-year income. More fine-grained and comprehensive measures of respondents’ financial situation than were available in these data might help clarify this relationship.

Finally, the association between holding multiple jobs and GDM was elevated among those who reported a Hispanic ethnic identity compared to single-job holders in the same racial/ethnic category. The association between holding multiple jobs and pregnancy-related hypertension was also elevated within this group compared to single-job holders in the same racial/ethnic group, with a similar magnitude odds ratio but less precision. Racial/ethnic identity might proxy disproportionate distribution into more or less favorable working conditions among multiple-job holders [51], driven by hiring bias, pass-over for promotions or elevation into non-temporary employment status, biased task distribution, lower control, and interpersonal discrimination in the workplace. The association between holding multiple jobs and maternal morbidities could also compound the effects of non-work-related inequities experienced by Hispanic pregnant workers in the U.S.

Our results did not suggest an association between changing jobs and GDM or pregnancy-related hypertension, either in a main effect association or in any of the stratified analyses conducted. Lower income, ever smoking during approximately the first trimester, and lower educational attainment were more common among job changers than single-job holders, yet none of our analyses suggested higher odds of GDM or pregnancy-related hypertension for job changers compared with single-job holders.

This finding could be due to heterogeneity within the “job changers” condition. We did not have access to information about motivations for job changes (e.g., whether respondents experienced involuntary job loss versus voluntarily changing jobs), nor whether job changes resulted in moves to more or less favorable working conditions. Disaggregating job changers by these characteristics could allow future researchers to explore whether job change may have a positive association with maternal health in favorable conditions, and whether job change in unfavorable conditions may have a negative association with health, as suggested by job loss’s inclusion in life stress indices in the prior literature [23].

Our analysis faced some limitations. Several factors such as access to job benefits (e.g., health insurance), subjective financial strain, detailed work scheduling information, and motivations for work patterns, had they been available in the data set, may have shed additional light on heterogeneity in the multiple job holding construct and possible mechanisms explaining its relationship to maternal morbidity. Additional information about socioeconomic position might be particularly useful because financial motivations may be associated with less beneficial multiple job holding [43,46], and because a single categorical income variable is an imperfect proxy for financial situation. Future studies may consider exploring additional potential mediators and moderators, including whether measures of perceived stress, scheduling pressures, physical activity, dimensions of employment quality, or other factors may explain part of the association between holding multiple jobs and maternal morbidity. Future studies may also consider assessing the distribution of occupation by working pattern to assess whether multiple-job holders or job changers are more likely to hold certain jobs compared to single-job holders. Finally, sample size limited our ability to analyze disaggregated racial/ethnic groups, though disparities in GDM and pregnancy-related hypertension differ by group [12]. Despite these gaps, NBDPS provided an important opportunity to study working pattern and maternal health, given its large sample of pregnant workers who were asked about not just one but all of the jobs they held during approximately one year leading up to delivery.

The study analyzed data collected between 1997–2011, and thus may not be representative of the current labor market in terms of the proportion of pregnant workers with multiple simultaneous jobs, or in terms of the distribution of employment quality across working patterns. There is some, though debated, evidence that holding multiple jobs and non-standard work arrangements may have increased since NBDPS data collection was completed [52], suggesting that continued study of these working patterns might be increasingly important.

## 5. Conclusions

Our findings suggest that asking about a single “main” job may not be adequate to understand the relationships between work and GDM or pregnancy-related hypertension. Future studies might benefit by obtaining information on the full complement of respondents’ jobs.

There are several policy- and practice-related implications of an association between holding multiple jobs and maternal morbidity. Workplaces are key sites of positive interventions that can support health; if the associations identified by this study are driven by poorer employment quality more common to jobs held simultaneously, such as non-standard work arrangements, then strengthening workplace protections and increasing employment quality for those jobs might contribute to reducing maternal morbidity.

Further, the results of our stratified analysis align with the hypothesis that respondents identifying with Hispanic or non-White racial/ethnic categories may be disproportionately sorted into multiple-job holding situations with less favorable employment quality, suggesting that research on employment quality and disparities in maternal health [11] could be explored further.

## Figures and Tables

**Table 1:** Distribution of variables among single-job holders, job changers, and multiple-simultaneous-job holders, National Birth Defects Prevention Study, 1997–2011.

	Single-Job Holders (n = 6917)		Job Changers (n = 554)		Multiple-Job Holders (n = 669)	
	*n*	*%*	*n*	*%*	*n*	*%*
Gestational diabetes mellitus	313	4.7	16	3.0	41	6.3
Missing ^1^	204	3.0	18	3.3	19	2.8
Pregnancy-related hypertension ^2^	242	9.0	15	7.4	35	12.8
Missing	152	5.3	13	6.0	10	3.5
Household income *						
Below 30,000 USD	2516	38.7	320	61.7	264	41.6
30,000 USD or above	3973	61.2	199	38.3	370	58.4
Missing	428	6.2	35	6.3	35	5.2
Peak weekly working hours *						
<32 h	1822	26.3	61	11.0	98	14.5
32–45 h	4141	59.9	354	63.9	152	22.7
>45 h	954	13.8	139	25.1	419	62.8
Maternal racial/ethnic category *						
Non-Hispanic White	4346	62.8	343	61.9	472	70.6
Non-Hispanic Black	767	11.1	92	16.6	90	13.5
Hispanic	1363	19.7	82	14.8	73	10.9
Other category	441	6.4	37	6.7	34	5.1
Maternal age at delivery **						
Years (mean, SD)	28.4	5.8	25.2	5.4	27.9	5.6
Maternal educational attainment *						
Less than high school	714	10.3	63	11.4	30	4.5
High school	1591	23.0	162	29.2	126	18.9
Some college	1977	28.6	193	34.8	239	35.8
College or more	2622	38.0	136	24.6	273	40.9
Missing	13	0.2	***		***	
Maternal BMI pre-pregnancy						
Underweight	315	4.7	34	6.2	23	3.5
Normal weight	3657	54.2	289	52.8	352	53.0
Overweight	1560	23.1	118	21.6	158	23.8
Obese	1216	18.0	106	19.4	131	19.7
Missing	169	2.4	7	1.3	5	0.8
Maternal smoking during pregnancy *						
Yes	1239	17.9	179	32.3	118	17.6
No	5676	82.1	375	67.7	551	82.4
Missing	***		***		***	
Maternal nativity *						
U.S. born	5721	82.8	500	90.3	602	90.0
Foreign born	1188	17.2	54	9.8	67	10.0
Missing	8	0.1	***		***	
Index pregnancy: Primigravida *	2188	31.6	213	38.5	263	37.2
Index pregnancy: Primipara *	3038	43.9	287	51.8	349	52.2
Missing	***		***		***	
Study Site *						
Arkansas	884	12.8	86	15.5	75	11.2
California	632	9.1	56	10.1	56	8.4
Iowa	843	12.2	85	15.3	134	20.0
Massachusetts	1027	14.9	18	3.3	40	6.0
New Jersey	376	5.4	15	2.7	17	2.5
New York	610	8.8	55	9.9	64	9.6
Texas	649	9.4	51	9.2	37	5.5
CDC/Atlanta	756	10.9	72	13.0	78	11.7
North Carolina	578	8.4	42	7.6	70	10.5
Utah	562	8.1	74	13.4	98	14.7

Variables with missing values include lines specifying missingness counts and percentage by working pattern. * Chi-square *p*-value is <0.05. ** *t*-test indicated that both job changers’ and multiple-simultaneous-job holders’ mean age significantly differed from that of single-job holders (*p* < 0.05). *** Cells with counts under 5 are suppressed. ^1^ Missing values include missing data as well as respondents whose self-reported diabetes diagnosis was not gestational diabetes, and/or whose diagnosis did not occur during the index pregnancy. ^2^ Pregnancy-related hypertension counts and proportions are drawn from a sample restricted to respondents interviewed after an updated survey launched in 2006.

**Table 2:** Associations between gestational diabetes or pregnancy-related hypertension and working pattern adjusted for maternal age and education, National Birth Defects Prevention Study, 1997–2011 *.

	Single-Job Holders		Job Changers		Multiple-Job Holders	
	Unadj.	Adj.	Unadj.	Adj.	Unadj.	Adj.
*Gestational diabetes mellitus* *OR (95% CI)*	n = 6713	n = 6713	n = 536	n = 536	n = 650	n = 650
	ref.	ref.	0.63 (0.38–1.05)	0.76 (0.45–1.27)	1.38 (0.98–1.93)	**1.53** **(1.09–2.14)**
*Pregnancy-related hypertension* *OR (95% CI)*	n = 2696	n = 2690	n = 204	n = 204	n = 273	n = 273
	ref.	ref.	0.81 (0.47–1.39)	0.79 (0.46–1.37)	**1.49** **(1.02–2.18)**	**1.53** **(1.04–2.24)**

* Bolded format indicates a statistically significant difference (α = 0.05) between multiple-job holders and single-job holders in the same stratum.

**Table 3:** Associations between gestational diabetes or pregnancy-related hypertension and working pattern, stratified by prior-year household income and adjusted for maternal age and education, National Birth Defects Prevention Study, 1997–2011 *.

	Single-Job Holders		Job Changers		Multiple-Job Holders	
*Prior-year household income strata*	≤30,000 USD	>30,000 USD	≤30,000 USD	>30,000 USD	≤30,000 USD	>30,000 USD
*Gestational diabetes mellitus* *aOR (95% CI)*	n = 2420	n = 3870	n = 313	n = 190	n = 257	n = 358
	ref.	ref.	0.68 (0.34–1.37)	0.72 (0.29–1.79)	**1.72** **(1.03–2.87)**	1.40 (0.86–2.26)
*Pregnancy-related* *hypertension* *aOR (95% CI)*	n = 1002	n = 1598	n = 127	n = 75	n = 107	n = 160
	ref.	ref.	0.54 (0.24–1.19)	1.32 (0.62–2.81)	1.40 (0.76–2.56)	1.61 (0.97–2.66)

* Bolded format indicates a statistically significant difference (α = 0.05) between multiple-job holders and single-job holders in the same stratum.

**Table 4:** Associations between gestational diabetes or pregnancy-related hypertension and working pattern, stratified by peak weekly working hours and adjusted for maternal age and education, National Birth Defects Prevention Study, 1997–2011 *.

	Single-Job Holders			Job Changers			Multiple-Job Holders		
*Peak weekly working hours strata*	<32 h	32–45 h	>45 h	<32 h	32–45 h	>45 h	<32 h	32–45 h	>45 h
*Gestational diabetes mellitus* *aOR (95% CI)*	n = 1762	n = 4010	n = 929	n = 58	n = 345	n = 133	n = 97	n = 146	n = 406
	ref.	ref.	ref.	0.52 (0.07–3.88)	0.69 (0.36–1.32)	0.86 (0.33–2.24)	0.85 (0.26–2.82)	**2.61** **(1.51–4.52)**	1.17 (0.69–2.00)
*Pregnancy-related hypertension* *aOR (95% CI)*	n = 723	n = 1566	n = 401	n = 32	n = 130	n = 42	n = 39	n = 67	n = 167
	ref.	ref.	ref.	0.36 (0.05–2.71)	0.57 (0.26–1.26)	1.65 (0.68–4.02)	2.07 (0.76–5.63)	1.15 (0.51–2.56)	1.43 (0.82–2.51)

* Bolded format indicates a statistically significant difference (α = 0.05) between multiple-job holders and single-job holders in the same stratum.

**Table 5:** Associations between gestational diabetes or pregnancy-related hypertension and working pattern, stratified by racial/ethnic category and adjusted for maternal age and education, National Birth Defects Prevention Study, 1997–2011 *.

	Single-Job Holders			Job Changers			Multiple-Job Holders		
*Racial/ethnic category*	Non-Hispanic White	Non-Hispanic Black	Hispanic (any race)	Non-Hispanic White	Non-Hispanic Black	Hispanic (any race)	Non-Hispanic White	Non-Hispanic Black	Hispanic (any race)
*Gestational diabetes mellitus* *aOR (95% CI)*	n = 4237	n = 748	n = 1299	n = 334	n = 90	n = 78	n = 456	n = 88	n = 73
	ref.	ref.	ref.	0.72 (0.33–1.57)	0.67 (0.20–2.23)	0.61 (0.19–2.01)	1.37 (0.85–2.21)	1.73 (0.78–3.86)	**2.25** **(1.09–4.65)**
*Pregnancy-related hypertension* *aOR (95% CI)*	n = 1621	n = 281	n = 596	n = 121	n = 28	n = 35	n = 188	n = 32	n = 33
	ref.	ref.	ref.	1.14 (0.62–2.10)	**	0.36 (0.05–2.72)	1.35 (0.84–2.19)	1.46 (0.53–3.98)	2.05 (0.75–5.60)

* Bolded format indicates a statistically significant difference (α = 0.05) between multiple-job holders and single-job holders in the same stratum. ** Model did not converge.

## Data Availability

The dataset presented in this article are not readily available to the public. Procedures to request access to the dataset can be reviewed at https://www.cdc.gov/ncbddd/birthdefects/nbdps-public-access-procedures.html (accessed on 5 March 2024).

## Supplementary Materials

The following supporting information can be downloaded at: https://www.mdpi.com/article/10.3390/ijerph21050619/s1, Table S1: Associations between gestational diabetes or pregnancy-related hypertension and working pattern, stratified by prior-year household income and adjusted for maternal age and education, National Birth Defects Prevention Study, 1997–2011. Income cut points are set at 10,000 USD and 50,000 USD*.
