# Supplementary material for: Multiple Job Holding, Job Changes, and Associations with Gestational Diabetes and Pregnancy-Related Hypertension in the National Birth Defects Prevention Study
Source: Int J Environ Res Public Health. 2024 May 14;21(5):619. doi: 10.3390/ijerph21050619 (PMC11121455; doi:10.3390/ijerph21050619)
Supplement: Supplementary file 1 [file ijerph-21-00619-s001.zip › ijerph-2926683-supplementary.pdf]

Supplementary Material

Multiple Job Holding, Job Changes, and Associations with Gestational Diabetes and Pregnancy-Related Hypertension in the National Birth Defects Prevention Study

Amel Omari [1,2,*], Miriam R. Siegel [1], Carissa M. Rocheleau [1], Kaori Fujishiro [1], Kristen Van Buren [1], Dallas Shi [1,2], A.J. Agopian [3], Suzanne M. Gilboa [4] and Paul A. Romitti [5]

[1] Division of Field Studies and Engineering, National Institute for Occupational Safety and Health, Cincinnati, OH 45213, USA
[2] Epidemic Intelligence Service Officer, Centers for Disease Control and Prevention (CDC), Atlanta, GA 30329, USA
[3] Department of Epidemiology, Human Genetics, and Environmental Sciences, UTHealth School of Public Health, Houston, TX 77030, USA
[4] Division of Birth Defects and Infant Disorders, National Center on Birth Defects and Developmental Disabilities, Centers for Disease Control and Prevention (CDC), Atlanta, GA 30329, USA
[5] Department of Epidemiology, College of Public Health, The University of Iowa, Iowa City, IA 52242, USA
* Correspondence: rlx9@cdc.gov

Table S1. Associations between gestational diabetes or pregnancy-related hypertension and working pattern, stratified by prior-year household income and adjusted for maternal age and education, National Birth Defects Prevention Study, 1997-2011. Income cut points are set at $10,000 and $50,000.*

	Single job holders			Job changers			Multiple job holders		
Prior-year household income strata	<$10K	$10K-50K	>$50K	<$10K	$10K-50K	>$50K	<$10K	$10K-50K	>$50K
Gestational diabetes aOR (95% CI)	n=887<br>ref.	n=2712<br>ref.	n=2733<br>ref.	n=126<br>1.05 (0.46-2.39)	n=282<br>0.67 (0.31-1.46)	n=97<br>0.28 (0.04-2.05)	n=67<br>1.06 (0.37-3.08)	n=322<br>**1.70 (1.04-2.77)**	n=227<br>1.56 (0.88-2.78)
Pregnancy-related hypertension aOR (95% CI)	n=415<br>ref.	n=1024<br>ref.	n=1173<br>ref.	n=52<br>0.86 (0.29-2.55)	n=107<br>0.58 (0.26-1.29)	n=44<br>1.26 (0.44-3.64)	n=25<br>**4.24 (1.64-10.94)**	n=136<br>0.74 (0.39-1.42)	n=107<br>**2.14 (1.20-3.84)**

*All models have been adjusted for maternal age and educational attainment. Bolded format indicates statistically significant difference (α=0.05) between multiple job holders and single job holders in the same stratum.
